# Fatigue, monocyte activation, and degree centrality of the thalamus in post-menopausal women living with HIV

**DOI:** 10.1007/s11682-025-01028-3

**Published:** 2025-06-16

**Authors:** Kaitlyn Dillon, Judith Lobo, Suresh Pallikkuth, Bonnie Levin, Roger McIntosh

**Affiliations:** 1 University of Miami, Coral Gables, FL USA; 2 University of California San Diego, San Diego, CA USA; 3 University of Miami Miller School of Medicine, Miami, FL USA

**Keywords:** Fatigue, HIV, Thalamocortical, Monocyte activation

## Abstract

Post-menopause is associated with chronic fatigue, inflammation, and aberrant brain connectivity, however there is a dearth of studies comparing these effects as a function of HIV. The current study investigated whether degree centrality of the thalamus mediates the effect of sCD14, a marker of monocyte activation, on fatigue and whether those parameters vary as a function of HIV-status. Resting-state functional connectivity MRI data, blood plasma, and self-report data were collected from 16 HIV + and 25 HIV- post-menopausal women. Analyses tested whether degree centrality of the thalamus, caudate, and right putamen mediated the relationship between sCD14 and fatigue. HIV-serostatus was then tested as a moderator. Compared to HIV-negative, HIV + women had higher levels of sCD14, *t*(34) = -3.85, *p* <.001, and lower thalamic degree centrality, *t*(33) = 2.17, *p* =.038. SCD14 predicted lower thalamic DC, *b* = -1.16, *p* =.035. Degree centrality predicted fatigue, *b* = -4.50, *p* =.03. Indirect and moderation effects were not significant. Monocyte activation is feature of chronic HIV-infection that impacts the number of thalamic connections to whole brain. The reduced thalamic degree centrality is associated with greater neurobiological vulnerability for fatigue after menopause.

The definition of fatigue is nebulous and may describe unpleasant cognitive and emotional symptoms of exhaustion, often accompanied by diminished functional capacity that doesn’t remit after a full night’s sleep (Barroso et al., 2015). Fatigue is among the symptoms most frequently reported by persons living with HIV (Barroso & Voss, 2013; Barroso et al., 2015; Sullivan et al., 2003), with estimated prevalence ranging from 33 to 88%. Although the experience of fatigue is heterogeneous among individuals with HIV, it has been linked to a reduction in socio-occupational functioning, poorer HIV disease management, worse global cognitive impairment in HIV + compared to HIV- individuals, and is among the strongest underlying factors of the frailty phenotype (Barroso & Voss, 2013; Falutz et al., 2021; Barroso et al., 2015; Campbell et al., 2022).

Investigations to identify biomarker predictors of fatigue have yielded inconsistent results for HIV disease markers such as CD4 count (Voss, 2005; Barroso et al., 2003). Chronic inflammation persists in older adults living with HIV and is not dependent on CD4:CD8 ratios or virologic suppression (Serrano-Villar et al., 2014). Additionally, this inflammation predicts non-AIDS-associated morbidities and mortality (Vujkovic-Cvijin et al., 2019; Aberg, 2012; Somsouk et al., 2015). Monocyte activation is an essential innate immune response, however maintenance of this pro-inflammatory response may drive pathogenesis in chronic disease (Knudsen et al., 2022). Soluble forms of monocyte surface proteins (sCD14 and sCD163) are shed by activated monocytes an hence suitable indicator of levels of peripheral activation (Knudsen et al., 2022). While the role of monocytes varies in disease models and with disease progression, monocyte activation has been linked to the presence of fatigue in several clinical syndromes such as chronic fatigue syndrome, long COVID, cirrhosis, and frailty (Chao et al., 1991; Albillos et al., 2006; Verschoor et al., 2014; Zhao et al., 2020). In a sample of patients who developed long COVID, fatigue symptom severity significantly correlated with increased intermediate and non-classical monocytes (Berentschot et al., 2023). Monocyte-related chemokines (e.g., MCP-1 and MIP-1Β) were associated with the frailty phenotype, a presentation underscored by elevated reports of fatigue (Su et al., 2017). In addition to its utility as a validated biomarker of monocyte activation, as well as microbial translocation, elevated sCD14 has also been linked to endotoxemia, intestinal barrier damage, and general gut dysbiosis in PLWH (Lien et al., 1998; Lv et al., 2021). In a study of comparing PLWH to people living without HIV (PLWOH), sCD14, alongside sCD163, another marker of monocyte activation, were among the most discriminant markers of inflammation, even after adjusting for age, smoking, alcohol use, and BMI (Novelli et al., 2020; Knudsen et al., 2022).

Fatigue is not only an aspect of chronic HIV disease management, but also a prominent symptom of menopause. In postmenopausal women living with HIV, fatigue remains one of the most burdensome symptoms reported, alongside muscle aches and pains and sleep disturbance (Schnall et al., 2018; Jehan et al., 2015). Some evidence indicates that menopause is associated with higher plasma sCD14 and sCD163 adjusting for age and other covariates, with sCD14 increasing during menopause transition (Peters et al., 2022; Shieh et al., 2020; Martin et al., 2013).

Despite the evidence supporting chronic inflammation as a common underlying factor for fatigue, it is unclear how these peripheral processes alter its presumed neurobehavioral underpinnings. Work across chronic inflammatory disease states has implicated the thalamus and subcortical basal ganglia in the manifestation of fatigue. In patients with multiple sclerosis for whom fatigue is common, disrupted functioning between the thalamus, basal ganglia, and cortex has been linked to manifestation of fatigue (de la Cruz et al., 2018). The thalamus in particular has been proposed as a prominent region in this pathogenesis, although the evidence characterizing these pathways appear to suggest compensatory activity increases as well as activity reductions (Capone et al., 2020). Additionally, these circuits appear sensitive to chronic inflammation. For example, neuroimaging abnormalities found within the striatum of the basal ganglia have been linked to fatigue in patients with chronic Hepatitis C, supporting the central manifestation of fatigue in inflammatory disease (Thames et al., 2015). While there is likely no single neural signature associated with fatigue, the thalamus’ role as a dynamic hub in modulating effort in response to many inputs establishes the thalamus as an important region in characterizing pathogenesis of fatigue. In examining the neural correlates of fatigue, the importance or centrality of key network nodes such as the thalamus may be a useful indicator of disruptions to functional connectivity in chronic disease states such as HIV (Rubinov et al., 2010).

Fatigue and monocyte activation are common features of chronic HIV disease management, particularly in post-menopausal women. Signals of inflammation are detected by afferent vagal pathways to the nucleus tractus solitarius and parabrachial nucleus before being relayed to the basal ganglia and then onto cortical regions involved in interoceptive processing (Goehler et al., 2000; Craig, 2002). While measures of monocyte activation have been linked to fatigue in other inflammatory-immune conditions, our understanding of the mediating role of the central nervous system is limited. Specifically, the role of aberrant thalamocortical connectivity in inflammation-driven fatigue is unclear, particularly in women living with HIV who may experience higher burden of chronic inflammation, monocyte activation, and fatigue. The current study seeks to specifically elucidate the mediating role of thalamic degree centrality on the relationship between sCD14 and fatigue symptoms in a sample of post-menopausal women. Additionally, we will explore the main and interactive effect of HIV status on sCD14 and thalamic degree centrality.

## Methods

### Participants

A community sample of post-menopausal women with no history of head trauma, stroke, coronary artery disease, Hepatitis C, cancer, diabetes (Type I or II), or neuropsychiatric disorders except depression or anxiety not treated with psychopharmacological therapies was recruited for a study approved by the Institutional Review Board at the University of Miami. Participants were considered post-menopausal if a minimum of one year had elapsed since their last menstrual period and if they were not receiving hormone replacement therapy. Participants living with HIV had undetectable viral load and stable ART. Additionally, participants tested negative for opiate, amphetamine, and cocaine use based on urine toxicology. Data from 41 participants was collected. Four participants were excluded for excessive head motion defined by an average volume of translational movements (≥ 3 mm in any direction) and one participant did not complete the resting-state scan.

Blood plasma samples were obtained between 0730 and 1000 h following 12 h of fasting and were stored for batch processing at -80 degrees Celsius. Functional MRI was performed on the same day (11% participants) or within approximately six months of the blood draw (56% participants within 14 days; *M* = 200.05 days, *SD* = 350.63). Blood draw and survey (e.g., BDI) data were collected on the same day. Participants were also asked to abstain from exercise and alcoholic beverages 24 h before the appointment and from drinking caffeinated beverages on the morning of the blood draw and fMRI session. The HIV-positive participants’ most recent CD4 count and HIV viral load results, date of HIV diagnosis, current ART regimen, and length of duration of ART treatment at the time of participation were extracted from their medical records, or self-report medical history were recorded if medical records were unavailable.

### fMRI imaging

#### Image acquisition

MRI data scans were collected on a 3.0 T Discovery MR750 GE scanner according to protocols approved by the institutional review board of the University of Miami. Each subject completed a 7-min resting-state fMRI scan with their eyes open. Resting-state scans were collected using an echo-planar imaging (EPI) sequence (Repetition time (TR)/TE = 2000/25 ms, flip angle = 90°, FOV = 24 mm × 24 mm, matrix = 96 × 96, slices = 42 thickness = 3 mm, 210 volumes). Prior to analysis, MRI data were visually inspected for warping, head coverage, blurring, ringing, striping, ghosting or signal loss (see https://www.fcon_1000.projects.nitrc.org/indi/enhanced/qc.html.

#### fMRI preprocessing

The fMRI data was preprocessed using the DPABI toolbox (Yan et al., 2016). Preprocessing included removing the first four volumes, realigning the images, and co-registering to T1 structural images. Given that head motion is a major contributor to noise during resting-state fMRI analyses (Satterthwaite et al., 2013), a Friston 24 correction with autoregressive models of motion was applied, incorporating 6 head motion parameters translated from x (pitch), y (roll), and z (yaw), one time point before, and the 12 corresponding squared items (Friston et al., 1996). This index of head displacement in 6 directions was recorded for each participant and later used for exclusion criteria. T1-weighted 3D images were segmented into modulated normalized parts of nuisance covariates (signals associated with gray matter, white matter, and cerebrospinal fluid). The normalized EPI volumes were re-sampled to a voxel size of 3 mm x 3 mm x 3 mm in MNI space. EPI images were then band-pass filtered at (0.008–0.08 Hz). These temporal filtering parameters were selected based on prior work demonstrating that spontaneous fluctuations upon which functional connectivity analyses are based exist in the range of 0.01–0.1 Hz (Biswal et al., 1995). The data was then spatially smoothed using a 6-mm full-width at half maximum (FWHM) isotropic Gaussian filter kernel and data scrubbing was implemented using linear interpolation.

#### fMRI region of interest

Two spatially distinct ROIs were used to extract an average hemodynamic time series over the course of the resting-state scan. These ROI spatial masks were based upon an independent component-derived basal ganglia network (Shirer et al., 2012). The spatial coordinates provided included approximately 204 voxels of the right thalamus, caudate and putamen (MNI: X = 14, Y = 2, Z = 10) and 247 voxels throughout the left thalamus and caudate (MNI: X = -24, Y = 4, Z = 8). The two functional ROIs containing the left and right thalamus and caudate as well as the right putamen were averaged for analyses.

### Measures

#### Fatigue

The Profile of Mood States (POMS) questionnaire was administered (Pollock et al., 1979) to assess fatigue. The POMS is a 65-item, Likert-scale rated self-report measure that assesses various mood states (e.g., fatigue-inertia). The fatigue subscale score was derived from the 7 items assessing subjective report of fatigue and related symptoms (score range 0–28). The Cronbach’s alpha for this subscale has been reported to range from 0.90 to 0.94 (OʼConnor et al., 2004). The POMS questionnaire has demonstrated acceptable reliability and good internal consistency when measuring fatigue across numerous populations (OʼConnor et al., 2004).

#### Degree centrality

Degree centrality is a network metric that quantifies the total number of links in a network that are connected to a given node. Mathematically, it can be presented as the formula for the sum of these links wherein *K*_*i*_ is the degree of node *i* and *a*_*ij*_ is the connection between nodes *i* and *j*.

$$\:{k}_{i}=\sum\:_{j=1}^{N}{a}_{ij}$$

A large value of degree centrality generally reflects the high importance or “hub-like” qualities of a given node in a network by virtue of the number of shared connections (van den Heuvel et al., 2013; Rubinov et al., 2010).

#### Plasma soluble SCD14

Plasma levels of sCD14 were determined using R&D systems Luminex Assay discovery panel (LXSAHM) in accordance with the manufacturer’s instructions. Plasma samples were thawed, vortexed, centrifuged at 1,000 x for 3 min, diluted 1:100 and incubated overnight using a mixture of beads for sCD14 with shaking. Subsequent washing was followed by incubation with biotinylated detection Abs for 1 h at room temperature. After Streptavidin-PE was added to the wells, an additional incubation period of 30 min followed at room temperature. Beads were subsequently washed and diluted with 150 µl Sheath Fluid prior to procurement using a MAGPIX instrument (Luminex Corporation). Mean Fluorescence Intensity (MFI) data were assessed via MILLIPLEX Analyst Software V.3.5 (EMD Millipore). Mean cytokine concentrations were resolved using standard curve and represented in ng/ml.

### Statistical analysis

Demographic, psychosocial, and biomarker data were compared between the HIV-positive and HIV-negative groups using an independent samples *t* test. Biomarker values were log_10_-transformed for group comparisons when values were found to exhibit a non-normal distribution. Mediation analyses were analyzed using the PROCESS macro (Hayes, 2013) for SPSS version 4.2 (models 4 and 7), conducted in SPSS version 27 (IBM Corp. Released 2020. IBM SPSS Statistics for Macintosh, Version 27.0. Armonk, NY: IBM Corp). Bootstrapped 95% confidence intervals were generated with 5000 iterations. Mediation was determined if confidence intervals of the indirect effects did not cross zero. A mediation model was conducted, wherein sCD14 levels were associated with fatigue, mediated by degree centrality of the averaged regions containing the bilateral basal ganglia network comprising the thalamus, caudate, and right putamen. A moderated mediation analysis (PROCESS macro model 7) was performed to examine the interaction effects of HIV status in the mediation model.

## Results

### Participants

Thirty-six participants (15 HIV+, 21 HIV-) were included in the study based on the availability of usable resting state fMRI scans, self-report mood surveys, as well as plasma markers of sCD14 data. As shown in Table 1, HIV + and HIV- participants did not differ significantly in age, race and ethnicity, months since menopause onset, body mass index, depression scores, or fatigue scores. As anticipated, HIV + participants (*M* = 6.25, *SD* = 0.09) showed significantly higher log-transformed sCD14 levels than did HIV- (*M* = 6.13, *SD* = 0.10), *t*(34) = -3.85, *p* <.001. HIV + participants (*M* = − 0.22, *SD* = 0.29) also showed smaller thalamic, caudate, and putamen degree centrality values compared to HIV- participants (*M* = 0.03, *SD =* 0.38), *t*(33) = 2.17, *p* =.038. Chronicity of HIV infection and long-term use of anti-retroviral therapy (ART) is reported in Table 2. A simple correlation was conducted to assess the relationship between fatigue and depression scores given their symptom overlap and they were highly correlated, *r* =.845, *p* <.001.

**Table 1:** Participant characteristics by HIV serostatus (*N* = 36)

Demographic variables	HIV- (*n* = 21)		HIV+ (*n* – 15)		Group comparison
	Mean (SD) or *n* (%)	Range	Mean (SD) or *n* (%)	Range	
Age	56.33 (6.84)	44–70	57.80 (4.68)	50–65	*t*(33.97) = -0.764, *p* =.450
Ethnicity					
Black White Hispanic or Latino/a	16 (76.19%) 1 (4.76%) 4 (19.05%)		12 (80%) 0 (0%) 3 (20%)		
sCD14 (log_10_ transformed)	6.12 (0.09)	5.89–6.28	6.25 (0.09)	6.09–6.39	*t*(34) = -3.85, *p* <.001*
Fatigue score	4.95 (4.40)	0–14	4.73 (3.96)	0–11	*t*(34) = 0.481, *p* =.634
Degree centrality of L region of interest	0.038 (0.438)	− 0.57–0.89	-0.25 (0.31)	− 0.84–0.27	*t*(33) = 2.12, *p* =.042*
Degree centrality of R region of interest	− 0.035 (0.353)	− 0.57 − 0.61	− 0.254 (0.298)	− 0.73 − 0.35	*t*(33) = 2.04, *p* =.05
Time since onset of menopause (months)	121.86 (104.68)	1–515	163.80 (158.97)	1–588	*t*(34) = − 0.447, *p* =.658
Body mass index	27.03 (5.19)	18.65–37.30	29.40 (5.97)	23.80–44.13	*t*(30) = -1.166, *p* =.253
Beck Depression Inventory scores	13.61 (12.73)	0–47	11.88 (12.5)	0–39	*t*(34) = 0.478, *p* =.636

Note. * denotes significance at *p* <.05

**Table 2:** HIV disease characteristics and treatment (*n* = 15)

Variable	Mean (SD) or *n* (%)
Persons reporting history of AIDS diagnosis	8 (19.5%)
Duration of HIV infection (yrs.)	18.82 (9.71)
Duration of exposure to ART (yrs.)	10.02 (8.18)
On nucleoside reverse transcriptase inhibitor (NRTI)	11 (73.2%)
On nucleotide reverse transcriptase inhibitor (NtRTI)	2 (4.9%)
On non-nucleoside reverse transcriptase inhibitors (NNRTI)	7 (17.1%)
On protease inhibitor (PI)	2 (4.9%)
On integrase inhibitor (II)	4 (9.8%)

Note. ART, antiretroviral therapy

### Mediation model

**Fig. 1:**
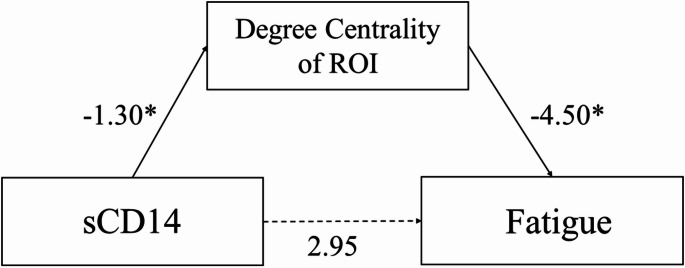
Mediation of thalamic region degree centrality on sCD14 and fatigue. Note. * denotes significance at *p* < .05

A mediation model was conducted, where sCD14 levels, a proxy of monocyte activation, predicted fatigue, mediated by degree centrality of the averaged regions containing the thalamus, caudate, and right putamen. The effect of sCD14 on degree centrality of the left thalamus, caudate, and putamen was negative and significant, *b* = -1.30, *SE* = 0.56, *p* =.035, with sCD14 accounting for 14.17% of variance in degree centrality, *R* =.38, *F*(1, 33) = 5.45. The effect of sCD14 on the right thalamus, caudate, and putamen These two predictors were significant in predicting fatigue, *R* =.42, *F*(2, 33) = 3.55, *p* =.04, accounting for 17.7% of variance in fatigue scores. When controlling for other predictors, degree centrality significantly predicted fatigue scores, *b* = -4.50, *SE* = 1.99, *p* =.03. Soluble CD14 did not significantly predict fatigue scores when controlling for degree centrality, *b* = 2.95, *SE* = 6.21, *p* =.64. Unstandardized pathways of this model are presented in Fig. 1. The indirect effect assessed whether degree centrality mediated the relationship between sCD14 and fatigue scores and was not significant, indirect effect = 5.22, *bootSE* = 3.21, *bootCI* [-0.11, 12.16]. The direct effect of sCD14 on fatigue scores was also not significant, direct effect = 2.95, *SE =* 6.21, *p* =.64.

### Moderated mediation model

**Fig. 2:**
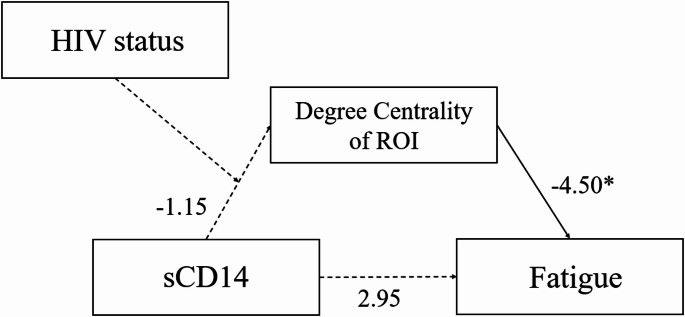
Moderation of HIV on mediation. Note. * denotes significance at *p* < .05

A moderated mediation analysis (PROCESS macro model 7) was performed to examine the interaction effects of HIV status in the mediation model tested above. The same criteria (i.e., confidence intervals excluding zero) were considered for significance. Neither serostatus (*b* = -5.00, *SE* = 7.94, *p* =.53), sCD14 (*b* = -1.15, *SE* = 0.81, *p* =.17), nor their interaction (*b* = 0.79, *SE* = 1.28, *p* =.54) significantly predicted degree centrality. Degree centrality significantly predicted fatigue, *b* = -4.50, *SE* = 1.99, *p* =.03 when controlling for sCD14, but sCD14 did not significantly predict fatigue when controlling for degree centrality, *b* = 2.95, *SE* = 6.21, *p* =.64. Unstandardized pathways of this model are presented in Fig. 2.

## Discussion

Despite adherence to ART medications, chronic fatigue and inflammation remain a feature of HIV disease. As expected, sCD14 was greater and degree centrality of the thalamus lower in PLWH. Given that fatigue is a prominent behavioral manifestation of chronic HIV infection, is it surprising that fatigue scores did not vary as a function of HIV status in our sample of postmenopausal women. Soluble CD14 was also expected to predict aberrant connectivity indexed by degree centrality of the thalamus, caudate, and putamen. Further, degree centrality of this region significantly predicted fatigue. HIV status did not moderate the relationship between sCD14, a marker of monocyte activation, and aberrant degree centrality of these regions. Factors such as BMI and ART use may have influenced our findings, as both have been shown to impact monocyte activation in PLWH. For example, in a study of PLWH on highly active ART, markers of monocyte activation were reduced after six months of highly active ART (Amiravan-Chevillard et al., 2000). In a study of virally suppressed PLWH, elevated BMI was independently associated markers reflective of monocyte activation and systemic inflammation (Conley et al., 2015). In our sample of virally suppressed PLWH, BMI appeared comparable to PLWOH, which may indicate that BMI-related inflammatory effects were present across both groups and may have minimized between-group differences. While the indirect effects of this mediation were not significant, these findings corroborate the role of monocyte activation in aberrant thalamic and basal ganglia connectivity, and the role of connectivity of these regions with the whole brain in fatigue.

In recent years, evidence has emerged showing that markers of peripheral inflammation directly subjugate the neural networks implicated in fatigue through mechanisms such as monoaminergic signaling. Given the extensive network of structures receiving dopaminergic innervation from the basal ganglia, inflammation can exert a wide negative impact on the connectivity of this structure with the rest of the brain (Labrenz et al., 2016; Dipasquale et al., 2016). Presynaptic striatal dopaminergic function is directly affected by signaling of inflammatory cytokines (e.g., IF-a). In a seminal study, altered activity in the basal ganglia was found following administration of endotoxin in the form of a typhoid vaccination (Capuron et al., 2012). In addition to these fMRI-based findings, PET data revealing increased inflammation was associated with decreased basal ganglia activity and corresponding behavioral alterations, including anhedonia, depression, and fatigue (Capuron et al., 2012). Inflammatory signaling from cytokines and activated immune cells in the periphery may subjugate the brain via the direct translocation through more vulnerable regions in the blood–brain barrier, afferent signaling to the vagus nerve, or humoral pathways (Vitković et al., 2000). A critical feature of this signaling pathway is the activation of enzymes that interfere with the production of essential monoaminergic cofactors, e.g., tetrahydrobiopterin, which are critically involved in the biosynthesis of dopamine as well as serotonin (Murr et al., 2002). Inflammatory insults can also modulate dopamine transporter function through a mitogen-activated protein kinase which can also lead to neuropsychiatric impairment (Morón et al., 2003). Thus, the basal ganglia is shown to be a target for the cytokine-based inflammation and inflammation-related changes in mood and sickness behavior predicated on malaise and fatigue (Felger et al., 2012).

The effect observed for thalamic degree centrality on fatigue is suggestive of a critical role for thalamic connectivity in the manifestation of fatigue. From a limited resource model perspective, the appraisal of fatigue can be viewed as waning level of commitment as a function of increased costs or decrease of available resources (Boksem et al., 2008; Baumeister et al., 2007). Evidence from task-based fMRI studies examining blood oxygen level dependent (BOLD) activity in relation to effortful task performance suggests increased connectivity of the thalamus with bilateral basal ganglia, cerebellum, and motor cortex during maximal versus minimally-induced muscular fatigue stages (Jiang et al., 2016). Jiang and colleagues further argued that increases in connectivity within the extended basal ganglia motor loop work to strengthen the descending central command to prolong effortful motor output during the waning of resources, which in turn signals the onset of fatigue (Jiang et al., 2016). One novel element of this study is the graph theory approach to quantifying functional connectivity between the thalamus ROI and whole brain. The observed association of lower degree centrality of the thalamus and higher fatigue implicates thalamocortical efficiency as a potential mechanism. A study examining whole-brain connectome changes in global network efficiency following administration of interferon-alpha showed reduced efficiency of information exchange within a thalamic/caudate node that corresponded with increased report of fatigue and other negative mood measures (Dipasquale et al., 2016).

Neuroimaging evidence suggests that PWH exhibit altered thalamic connectivity and other cortical-subcortical disruptions (Liŭ et al., 2020). For example, PLWH taking highly active antiretroviral therapies exhibited lower degree centrality of the left thalamus (Wang et al., 2023). A study using Bayesian estimates of rsFC between subcortical regions of interest showed that the caudate and thalamus appear to be regions illustrating the strongest difference between PLWH and healthy controls (Janssen et al., 2017). Structural changes to these regions have also been observed in PLWH, including gray matter volume deficits in the basal ganglia and thalamus (O’Connor et al., 2023) extending to the right calcarine, right cuneus, left hippocampus and parahippocampus (Liŭ et al., 2020). These findings yield evidence implicating lower thalamic degree centrality for persons with compared to those living without HIV. Neuroinflammation is well-characterized in PLWH evinced by disruption of long-distance connections throughout several major resting-state brain networks (Abidin et al., 2018; Thomas et al., 2015). Dopaminergic signaling pathways are already compromised in HIV and are susceptible to subtle measures of low-grade inflammation, including sCD14 implicated in our study. Despite this compelling evidence, we did not observe an interactive effect for HIV + status and thalamic DC on fatigue despite there being lower degree centrality in HIV + compared to HIV-negative individuals.

### Limitations

Our findings should be considered in light of several limitations. First, given the nature of resting-state fMRI studies, our sample size was small. FMRI researchers typically recommend large sample sizes to yield sufficient power (Jockwitz et al., 2021). The small sample and group size may limit the power of detecting significant effects, perhaps explaining the surprising finding of nonsignificant effect of HIV serostatus on the association between sCD14 and C_D_ of the ROI. While the small sample size and group stratification present statistical limitations, this sample size appears consistent with other clinical fMRI studies (Szucs & Ioannidis, 2020). Additionally, high population level power may not be necessary given that the study sought to characterize neural correlates of chronic HIV and menopause symptoms. Additionally, the use of bootstrapping in the mediation analyses reflects a default approach by the PROCESS macro for estimating indirect effects. Given the small sample size, the use of bootstrapping may raise concerns about potential bias. To address this concern, additional analyses were run using 5,000 samples, 1,000 samples, and without bootstrapping. The results remained consistent regardless of approach, and 5,000 samples were reported in the results above. The sample consisted of postmenopausal women with and without HIV and may not be generalizable to men or younger women living with or without the disease. Additionally, depression and fatigue are comorbid phenotypes with overlapping symptoms (Corfield et al., 2016), and indeed were highly correlated in our sample. While the women in our sample reported mild to moderate scores on the BDI and who denied current psychopharmacological treatment and thus may not be generalizable to individuals with a history of major depressive disorder or to those undergoing psychiatric treatment. Third, while our findings depict a path from the periphery to the brain that is empirically supported by previous studies (Rich et al., 2020), our findings are cross sectional and do not allow us to determine temporal precedence. Future research may seek to confirm causal relationships between monocyte activation, neural signatures of thalamic and BGN inefficiency, and fatigue. Additionally, the degree to which participants exercise may be associated with inflammation, fatigue, and thalamic function. Data assessing exercise frequency, intensity, and duration were not collected in the current study and should be considered in future work assessing monocyte activation, fatigue, and thalamic functioning. Given our conceptualization of fatigue as exhaustion and diminished functional capacity that doesn’t remit after a full night’s sleep, the absence of available sleep data for the current sample reflects another limitation. Future work may benefit from measurement of self-report fatigue symptoms alongside sleep quality data in order to rule out the effects of sleep disturbance.

## Conclusion

For ART-adherent HIV + women with advancing age, fatigue is a common, debilitating, and often neglected behavioral symptom. Our data suggest that elevated monocyte activation may contribute to patterns of aberrant connectivity in a basal ganglia network critical to interoceptive signaling and voluntary motor control. These patterns of connectivity may speak to reduced efficiency of thalamic nodes within a basal ganglia network poised to support motivated behavior. Our findings showing lower degree centrality of thalamic nuclei in PWH suggest a neurobiological vulnerability for fatigue after the onset of menopause. Overall, our findings contribute to a larger body of literature which implicate low-grade inflammation in the subjugation of neural networks that inform the representation of subjective fatigue, albeit in postmenopausal age women living with or without chronic HIV. This study may provide an important first step in assessing the biotype that underscores chronic fatigue in PWH.

## Data Availability

Data may be made available upon request to the corresponding author.
